# Developing global climate anomalies suggest potential disease risks for 2006 – 2007

**DOI:** 10.1186/1476-072X-5-60

**Published:** 2006-12-28

**Authors:** Assaf Anyamba, Jean-Paul Chretien, Jennifer Small, Compton J Tucker, Kenneth J Linthicum

**Affiliations:** 1 Goddard Space Flight Center, Biospheric Sciences Branch, Code 614.4, Greenbelt, MD 20771, USA; 2 Department of Defense Global Emerging Infections Surveillance & Response System, Division of Preventive Medicine, Walter Reed Army Institute of Research, 503 Robert Grant Avenue, Silver Spring, MD, USA; 3 USDA-Center for Medical, Agricultural & Veterinary Entomology, 1600/1700 S.W. 23rd Drive, Gainesville, Florida 32608, USA

## Abstract

**Background:**

El Niño/Southern Oscillation (ENSO) related climate anomalies have been shown to have an impact on infectious disease outbreaks. The Climate Prediction Center of the National Oceanic and Atmospheric Administration (NOAA/CPC) has recently issued an unscheduled El Niño advisory, indicating that warmer than normal sea surface temperatures across the  equatorial eastern Pacific may have pronounced impacts on global tropical precipitation patterns extending into the northern hemisphere particularly over North America. Building evidence of the links between ENSO driven climate anomalies and infectious diseases, particularly those transmitted by insects, can allow us to provide improved long range forecasts of an epidemic or epizootic. We describe developing climate anomalies that suggest potential disease risks using satellite generated data.

**Results:**

Sea surface temperatures (SSTs) in the equatorial east Pacific ocean have anomalously increased significantly during July – October 2006 indicating the typical development of El Niño conditions. The persistence of these conditions will lead to extremes in global-scale climate anomalies as has been observed during similar conditions in the past. Positive Outgoing Longwave Radiation (OLR) anomalies, indicative of severe drought conditions, have been observed across all of Indonesia, Malaysia and most of the Philippines, which are usually the first areas to experience ENSO-related impacts. This dryness can be expected to continue, on average, for the remainder of 2006 continuing into the early part of 2007. During the period November 2006 – January 2007 climate forecasts indicate that there is a high probability for above normal rainfall in the central and eastern equatorial Pacific Islands, the Korean Peninsula, the U.S. Gulf Coast and Florida, northern South America and equatorial east Africa. Taking into consideration current observations and climate forecast information, indications are that the following regions are at increased risk for disease outbreaks: Indonesia, Malaysia, Thailand and most of the southeast Asia Islands for increased dengue fever transmission and increased respiratory illness; Coastal Peru, Ecuador, Venezuela, and Colombia for increased risk of malaria; Bangladesh and coastal India for elevated risk of cholera; East Africa for increased risk of a Rift Valley fever outbreak and elevated malaria; southwest USA for increased risk for hantavirus pulmonary syndrome and plague; southern California for increased West Nile virus transmission; and northeast Brazil for increased dengue fever and respiratory illness.

**Conclusion:**

The current development of El Niño conditions has significant implications for global public health. Extremes in climate events with above normal rainfall and flooding in some regions and extended drought periods in other regions will occur. Forecasting disease is critical for timely and efficient planning of operational control programs. In this paper we describe developing global climate anomalies that suggest potential disease risks that will give decision makers additional tools to make rational judgments concerning implementation of disease prevention and mitigation strategies.

## Background

The El Niño/Southern Oscillation (ENSO) is the most well-known phenomenon influencing the global climate variability at interannual time scales. The National Oceanic and Atmospheric Administration's (NOAA) Climate Prediction Center (CPC) has recently issued an unscheduled El Niño conditions advisory that indicates that El Niño conditions will peak during the Northern Hemisphere winter, followed by weakening during March – May 2007 [1]. The term El Niño refers to the large-scale ocean-atmosphere climate phenomenon linked to a periodic warming in sea surface temperatures across the central and east-central equatorial Pacific (between approximately the International Date line and 120 degrees west longitude), and thus represents the warm phase of the ENSO, and is sometimes referred to as a Pacific warm episode. The opposite of which is La Niña, a cold phase of ENSO. Given the large size of the Pacific Ocean, changes in the sea surface temperature patterns and gradients across the basin influence atmospheric circulation with pronounced impacts on global tropical precipitation and temperature patterns.

Climate variability has a demonstrated impact on infectious diseases [2], and increased disease transmission has been linked to ENSO driven climate anomalies [3-7]. Outbreaks of insect transmitted diseases such as Murray Valley encephalitis, bluetongue, Rift Valley fever (RVF), African Horse sickness, Ross River virus disease [8-12] and malaria [13,14] have been associated with El Niño. Hence, forecasting the risk of ENSO related human and animal disease outbreaks is critical for timely and efficient planning of operational control programs. However, for decision makers to respond effectively the forecast must be accurate and timely [5]. Here we describe developing global climate anomalies that suggest potential elevated disease risks in the hope that decision makers will have additional tools to make rational judgments concerning implementation of a wide-range of disease mitigation strategies.

## Results

### Current and forecasted climatic conditions

**Figure 1:**
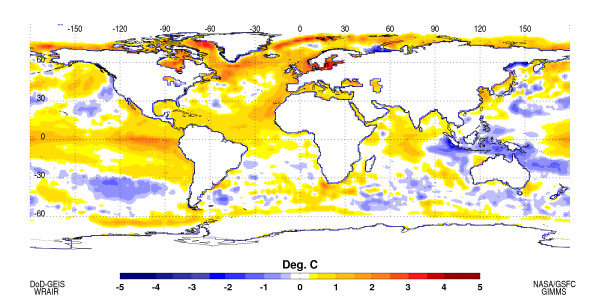
Sea Surface Temperature (SST) anomalies for October 2006. Above normal SSTs have developed in the equatorial eastern Pacific Ocean (~2°C) and also in the equatorial Indian Ocean (~1°C) typical of warm ENSO events. The SST anomalies are computed with respect to 1982–2006 base period means.

The NOAA CPC advisory indicates El Niño conditions have developed in the tropical Pacific and are likely to continue into early 2007. Sea surface temperatures (SST) increased remarkably in the equatorial Pacific during September and October 2006 (Figure 1).

**Figure 2:**
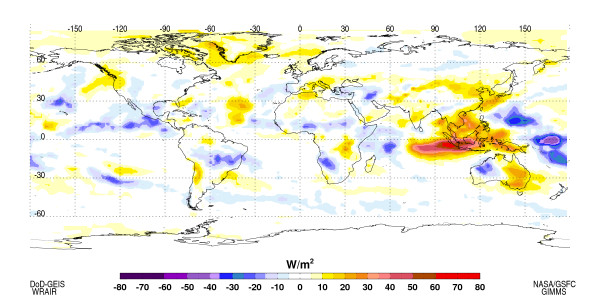
Outgoing Longwave Radiation (OLR) anomalies for October 2006. OLR anomalies are used to infer tropical precipitation. As shown here positive OLR anomalies indicate suppressed convection and very dry conditions (brown to red colors) over Western Pacific – Indonesian region and positive OLR anomalies illustrated enhanced precipitation in the central and eastern Pacific Ocean, western equatorial Indian Ocean, East Africa and across the Sahel (shown in shades of blue)

Some impacts from the developing El Niño are already evident in the pattern of tropical precipitation. During the October 2006, positive Outgoing Longwave Radiation (OLR) anomalies (suppressed convection and precipitation) conditions have been observed across all of Indonesia, Malaysia and most of the Philippines, which are usually the first areas to experience ENSO-related impacts. This dryness can be expected to continue, on average, for the remainder of 2006 continuing into the early part of 2007. In contrast negative OLR anomalies indicative of enhanced convection and precipitation were observed eastwards between the date line and Papua/New Guinea, and to the west in the equatorial western Indian Ocean (WIO) region extending into equatorial East Africa (Figure 2).

**Figure 3:**
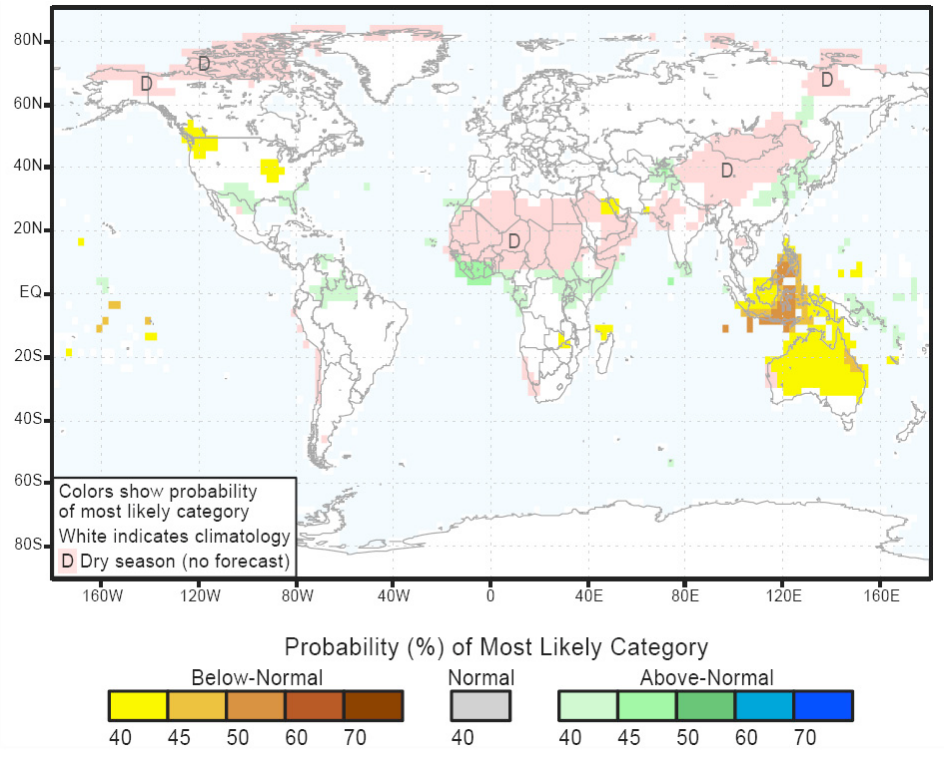
IRI Multi-Model Probability Forecast for Precipitation for November-January 2007, Issued September 2006.

For the forecast period season November 2006 – January 2007 [15] there is a high probability for above normal rainfall in the central and eastern equatorial Pacific Islands, the Korean Peninsula, the U.S. Gulf Coast and Florida, northern South America and equatorial Africa (Figure 3). In contrast, there is a high probability for drought conditions over Australia and the Indonesian Basin.

**Figure 4:**
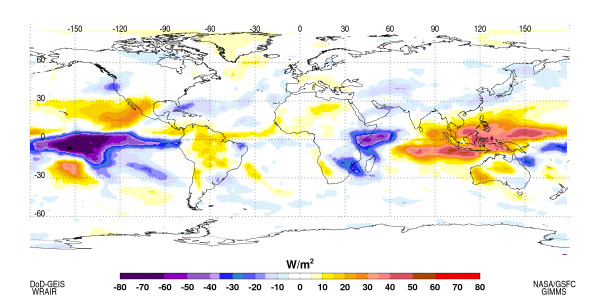
OLR anomalies at the peak of the 1997/98 El Niño showing suppressed convection and drought conditions over southeast Asia, Amazonia, western Australia and enhanced convection and precipitation over central and eastern Pacific Ocean, Peru, and Eastern Africa.

Using the 1997/98 period as a reference template (Figure 4) and the forecast of likely conditions for the next 3–9 months, there is a high likelihood for drought conditions to prevail over south-east Asia, Mexico, north-east Brazil and Southern Africa, and above normal rainfall and flood conditions to occur over coastal Peru, southern California, the U.S. Gulf Coast and Florida and Eastern Africa.

### Potential elevated disease outbreaks

**Figure 5:**
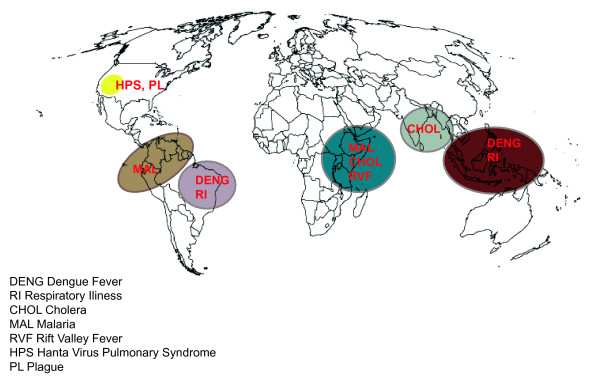
Hotspots of potential elevated risk for disease outbreaks under El Niño conditions: 2006 – 2007.

Some of the above climate extremes are already being experienced in equatorial East Africa, Australia and the Indonesian Peninsula. These extremes in climatic conditions will likely affect vector abundance in different ways elevating the risk of outbreaks of various infectious diseases [3]. Drought conditions can suppress predators of *Anopheles *malaria vectors [14,16]; however, heavy rains will boost food supplies – a synergy that can for example elevate rodent populations [17] and create appropriate conditions for mosquito breeding and propagation [11]. Previous ENSO events have been strongly associated with disease outbreaks over time and with spatial clusters of mosquito-, water and rodent-borne illnesses. Given current observations and forecast information the following regions (Figure 5) are at increased risk for disease outbreaks.

1. Indonesia, Malaysia, Thailand and most of the Southeast Asia Islands: Increased dengue fever transmission caused by drought conditions which (1) increase water storage around houses leading to elevated *Aedes aegypti *populations and (2) elevate ambient air temperatures which will reduce the extrinsic incubation period for the virus in vector mosquitoes increasing vector capacity [[18], Linthicum et al., unpublished observations]; increased respiratory illness due to haze from uncontrolled burning of tropical forests when extreme drought occurs.

2. Coastal Peru, Ecuador, Venezuela, and Colombia: Increased risk of malaria due to elevated *Anopheles *vector populations which will develop when various types of immature habitats are flooded after heavy rainfall follows a period of drought [14,16].

3. Bangladesh and coastal India: Elevated risk of cholera due to elevated sea surface temperatures and of incursion of plankton-laden water inland rich in *Vibrio cholerae*, the bacterium that causes cholera [7]. In addition to elevated SSTs, heavy rains wash nutrients into waterways and may trigger plankton blooms.

4. East Africa (Ethiopia, Kenya, Somalia, and Uganda): Increased risk for RVF and malaria resulting from elevated mosquito vector populations, and cholera caused by flooding due to heavy rainfall in dry land areas [7,11,19,20].

5. South West USA (New Mexico, Arizona): Increased risk for hantavirus pulmonary syndrome and plague due to elevated rodent populations caused by heavy rainfall [17,21]

6. Southern California: Elevated potential for transmission of arboviruses, such as West Nile virus, caused by heavy rainfall and resulting in elevated *Culex *species mosquito populations.

7. Northeast Brazil: Drought conditions leading to increased dengue fever and respiratory illness.

## Discussion

Currently weak El Niño conditions exist, but there is a potential for this event to strengthen into a moderate event by winter. The U.S. Department of Defense Global Emerging Infections Surveillance and Response System (DOD-GEIS) RVF Monitor website [22] publishes the current suite of products for monitoring El Niño conditions and implications for RVF activity in Africa and the Arabian Peninsular region. The global products including SSTs and OLR (a proxy indicator for rainfall) are useful in illustrating the current situation of global climate anomalies with implications for public health.

The development of weak El Niño conditions also helps explain why this Atlantic hurricane season has been less active than was previously expected. El Niño usually acts to suppress hurricane activity by increasing the vertical wind shear over the Caribbean Sea region. Typical El Niño effects are likely to develop over North America during the upcoming winter season. These include warmer-than-average temperatures over western and central Canada, and over the western and northern United States. Wetter-than-average conditions are likely over portions of the U.S. Gulf Coast and Florida, while drier-than-average conditions can be expected in the Ohio Valley and the Pacific Northwest.

The forecast extreme patterns are similar to the observed events during the 1997/98 El Niño event and similar such events in the past and are likely to have a significant impact on disease vectors, although the magnitudes of the anomalies are unknown. While all ENSOs do not behave alike (particularly in terms of teleconnections), and climate change and warmer SSTs overall may be altering ENSOs and thus exaggerating the droughts and rains where they occur, typical El Niño effects that are likely to develop during the period November 2006 – March 2007 include: drier-than-average conditions over most of Malaysia, Indonesia, some of the U.S.-affiliated islands in the tropical North Pacific, northern South America and southeastern Africa, and wetter-than-average conditions over equatorial East Africa, central South America (Uruguay, northeastern Argentina, and southern Brazil) and along the coasts of Ecuador and northern Peru. Over North America the upcoming winter season, is likely exhibit warmer-than-average temperatures over western and central Canada, and over the western and northern United States, wetter-than-average conditions over the U.S. Gulf Coast and Florida, and drier-than-average conditions in the Ohio Valley and the Pacific Northwest. In October 2006, drier-than-average conditions have been observed across all of Indonesia, Malaysia, most of the Philippines, and eastern Australia, and enhanced precipitation over the eastern Pacific Islands, portions of Central America and semi-arid equatorial eastern Africa.

The development of El Niño conditions has important implications for global public health. The dramatic displacements in large scale precipitation centers across the global tropics will likely lead to extremes in climate events with above normal rainfall and flooding in some regions and extended drought periods in other regions.

## Conclusion

An El Niño conditions advisory has been issued by the NOAA CPC which indicates that anomalously warm SST conditions exist in the equatorial Pacific in October 2006 and are likely to continue into early 2007 [1]. Global products including SST and OLR, which are useful in illustrating the current situation of global climate anomalies, are being monitored for their implications for public health [22]. Impacts of the current El Niño include above normal precipitation over the eastern Pacific and East Africa regions, and drier than average conditions over Southeast Asia, Malaysia and Indonesia. These conditions will likely persist for the remainder of 2006 and early 2007.

The development of El Niño conditions has significant implications for global public health. Extremes in climate events with above normal rainfall and flooding in some regions and extended drought periods in other regions will occur. Forecasted elevated rainfall in coastal Peru, Ecuador, Venezuela and Columbia will increase malaria risk due to elevated *Anopheles *vector populations. Heavy rainfall in East Africa may elevate mosquito vector populations and lead to RVF, and increased malaria and cholera risk. Elevated rainfall in the south west of the U.S. will increase the risk of the rodent-borne diseases hantavirus pulmonary syndrome and plague. Above normal rainfall in southern California will elevate the risk of West Nile virus. Elevated sea surface temperatures near Bangladesh and India will increase the risk of cholera. Drought conditions in Southeast Asia and islands of Indonesia and northeast Brazil will increase the risk of dengue fever and respiratory diseases.

Forecasting epidemics or epizootics is critical for timely and efficient planning of operational control programs if the forecast is accurate and delivered in a timely manner. In this paper we describe developing global climate anomalies that suggest potential disease risks so that decision makers will have supplemental tools to make rational judgments concerning implementation of a wide-range of disease prevention and mitigation strategies.

## Methods

We have used current and forecasted ENSO induced climate anomaly patterns and compared to the 1997–1998 ENSO event to infer potential disease risks for the end of 2006 and beginning of 2007. The 1997–1998 El Niño was the largest and warmest to develop in the Pacific Ocean in the past 100 years, and milestone for seasonal forecasting. The occurrence of ENSO phenomena results in shifts in precipitation patterns over the global tropics as illustrated below. Warm ENSO events are exemplified by above normal Sea Surface Temperatures (SSTs) in the Eastern Pacific (EP) and sometimes above normal SSTs in the Western Indian Ocean (WIO). Warm ENSO events are known to increase precipitation in some regions of such as the eastern Pacific Ocean islands and the Peruvian coast, US Southwest and equatorial East Africa, and result in droughts in other areas for example the Western Pacific region (Indonesian, Philippines), Australia, northeast Brazil and southern Africa [23-27].

Global scale precipitation anomalies, as described above, can be inferred from OLR data, for example as shown in the OLR anomalies for December 1997. OLR is an indicator of both how warm the earth's surface is and how clear the atmosphere is overhead. Warm surfaces radiate more in the longwave range, while low values of OLR are typically due to clouds in the atmosphere and the lowest values can be used to infer deep convective clouds.

The current seasonal forecasts from the International Research Institute (IRI) for Climate and Society at Columbia University [15] issued September and updated in October 2006 were used in this analysis.

The unscheduled El Niño conditions advisory issued by the National Oceanic and Atmospheric Administration's (NOAA) Climate Prediction Center (CPC) that indicates that El Niño conditions will peak during the Northern Hemisphere winter, followed by weakening during March – May 2007 [1] was used to form the genesis of this paper.

Anomalous climatic conditions caused by ENSO are now recognized to be linked with outbreaks of various human and animal diseases in various countries [28]. The eco-climatic conditions associated with disease outbreaks can now be effectively monitored using satellite data as illustrated here.

## Competing interests

The author(s) declare that they have no competing interests.

## Authors' contributions

AA, JS, JPC, CJT and KJL conceived the idea, interpreted the data, and drafted the manuscript. AA and JS developed the analyzed the data and created the graphics.
